# The relationship among motivation, self-monitoring, self-management, and learning strategies of MOOC learners

**DOI:** 10.1007/s12528-021-09301-2

**Published:** 2021-11-02

**Authors:** Meina Zhu, Min Young Doo

**Affiliations:** 1 Learning Design and Technology, Wayne State University, 365 Education Building, 5425 Gullen Mall, Detroit, MI 48202 USA; 2 Department of Education, College of Education, Kangwon National University, Chuncheon-si, Korea

**Keywords:** Self-directed learning, Learning strategies, Motivation self-monitoring, Self-management, MOOCs

## Abstract

In massive open online learning courses (MOOCs) with a low instructor-student ratio, students are expected to have self-directed learning abilities. This study investigated the relationship among motivation, self-monitoring, self-management, and MOOC learners’ use of learning strategies. An online survey was embedded at the end of three MOOCs with large enrollments asking for learners’ voluntary participation in the study. The survey results from 470 participants indicated that motivation positively influenced self-monitoring, self-management, and learning strategies. In addition, self-monitoring and self-management did not affect the utilization of learning strategies. This underscores learners’ motivation and the need to encourage them to adopt appropriate learning strategies for successful learning. The results also revealed that self-monitoring positively affected self-management. The findings highlight the critical need to enhance self-monitoring skills to further promote self-management skills in MOOCs. In addition, self-monitoring and self-management did not encourage learners to use related learning strategies in this study. This study should be extended to investigate practical ways to encourage MOOC learners to adopt learning strategies.

## Introduction

Massive open online courses (MOOCs), which were first introduced in 2008, give open access to learners around the world (Milligan & Littlejohn, 2016), with more than 900 universities providing over 11,000 MOOCs (Shah, 2019). Class Central (a website that tracks MOOC learning platforms) indicated that MOOCs have rapidly and significantly increased since March 2020 due to the enforcement of social distancing rules during the COVID19 pandemic (Rindlisbacher, 2020; Schaffhauser, 2020). Students who were not allowed to attend face-to-face classes often sought learning opportunities in MOOCs as an alternative to traditional courses. For example, students enrolled in over 10 million courses in Coursera in a 30-day period in 2020, representing a 644%increase (Schaffhauser, 2020).

MOOCs have different characteristics than traditional online courses, including tuition, credits, and the number of students who enroll (Pappano, 2012). MOOCs are usually free or have a low-cost fee for certificates or degrees compared to traditional education. In addition, a large number of students typically enroll in a MOOC with as many as 100,000 students per class. Consequently, student-instructor interaction is limited in MOOCs. In addition, students have more control over their own learning in MOOCs, including self-directed learning strategies and a flexible time and place to learn. However, the sudden transition to more learning control from the instructor to the learners poses challenges for learners (Fournier et al., 2014). In particular, learners need self-directed learning (SDL), where they take responsibility for their own learning (Lee et al., 2020).

Brookfield (2013) stated that self-directed learners could select the topic, learning strategies, and amount of content they want to learn as well as how to evaluate their own learning. SDL has been identified as a critical skill in diverse education settings (Hiemstra, 1994; Owen, 2002) and is an essential feature for lifelong learning (Dynan et al., 2008; Hyland & Kranzow, 2011; Sze-yeng & Hussain, 2010). Although SDL benefits learners in many ways (Sze-yeng & Hussain, 2010), including improving academic performance (Cleary & Zimmerman, 2004), the typically low instructor-to-student ratio in MOOCs underscores the importance of MOOC learners' SDL (Kop, 2011; Kop & Fournier, 2010; Rohs & Ganz, 2015).

Another pivotal component for successful online learning is whether to adopt appropriate learning strategies for learning. Kovanović et al. (2015) and Shen et al. (2013) noted that students tended to underuse appropriate learning strategies and tools for learning in online learning environments. Thus, it is critical to explore whether students’ learning strategies are affected by students’ SDL skills.

While SDL is a requirement for MOOC learners' successful learning, and appropriate learning strategies are important in online learning, limited research has examined student SDL skills and learning strategies in MOOCs. Therefore, the purpose of the present study is to examine the structural relationship among learning strategies in MOOCs and three key features of SDL: motivation, self-monitoring, and self-management. The research question guiding this study is, “To what extent do MOOC learners’ motivation, self-monitoring, and self-management skills predict their use of learning strategies?” The research findings and implications are expected to encourage MOOC learners to utilize learning strategies for successful learning.

## Literature review

### The development of self-directed learning

Tough (1971) first proposed that SDL was rooted in adult education (Merriam et al., 2007). There are two interpretations of SDL: as learners’ personal attributes and as a learning process (Brockett & Hiemstra, 1991). Researchers who have emphasized the personal attributes interpretation included Guglielmino (1978), Long (1991), and Merriam (2001). Long (1991) identified independence, self-efficacy, metacognitive awareness, intrinsic motivation, deep learning, and priority in learning as requirements for SDL. More recently, Sze-Yeng and Hussian (2010) added learner autonomy, which is the ability to control the learning process through personal responsibility as a personal attribute for SDL. Specifically, learners have the freedom to choose their behaviors (Deci & Ryan, 2008), which motivates them to engage in their own learning (Skinner et al., 2008). Similarly, Brookfield (2013) stated that self-directed learners should choose the topics they want to learn, the learning strategies to use, the amount of time to learn, and how they want to evaluate the results of their learning. The criticism of the personal attribute perspective is that it underestimates the influence of the external environment on learning (Ainoda et al., 2005) and thus may lead to a misunderstanding that SDL is solely determined by personal attributes.

The interpretation of SDL as a learning process is used in the present study. Knowles (1975) described SDL as “a process in which individuals take the initiative, with or without the help of others, in diagnosing their learning needs, formulating learning goals, identifying human and material resources for learning, choosing and implementing appropriate learning strategies and evaluating learning outcomes” (p. 18). Similarly, Brookfield (1986) viewed SDL as a process that allows learners to work independently or collaboratively to plan, implement, and evaluate their own learning.

**Fig. 1:**
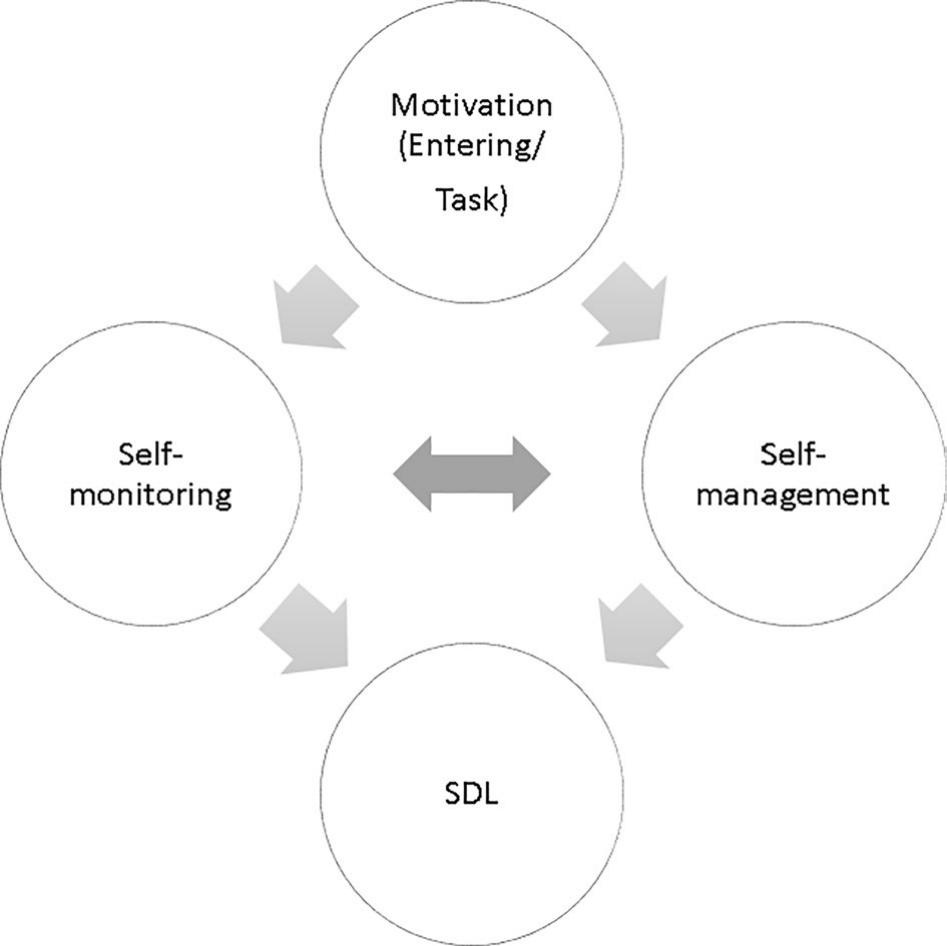
Self-directed learning model (Garrison, 1997)

Garrison (1997) described three interrelated aspects of SDL: (1) self-monitoring, (2) self-management, and (3) motivation (see Fig. 1). Self-monitoring is related to learners’ cognitive and metacognitive processes. According to Garrison (1997), self-monitoring refers to learners’ responsibility for the construction of personal learning, including cognitive and metacognitive processes. The self-management aspect focuses on the external environment and activities affecting the learning process. Learners should be able to manage their learning time as well as learning resources and support. The third aspect, motivation, is a predictor of learners’ behaviors and learning performance (Williams & Deci, 1996; Williams et al., 1997). It consists of initiating motivation (e.g., encouraging learning initiatives) and task motivation (e.g., learning persistence).

These three dimensions of SDL are interrelated (Garrison, 1997). For instance, the Zhu et al. (2020) confirmed that MOOC learners’ cognitive and meta-cognitive activities (i.e., self-monitoring) impacted their self-management. Abd-El-Fattah (2010) also investigated the relationship among motivation, self-monitoring, and self-management with 119 undergraduates in a face-to-face learning setting. The results of path analysis indicated that the three dimensions were interrelated, and motivation mediated the relationship between self-management and self-monitoring. Given that MOOCs require learners to be self-directed learners for successful learning outcomes, it is necessary to further examine the relationship among motivation, self-monitoring, and self-management for MOOC learners to provide practical implications for MOOC instructors and learners.

### SDL in traditional online courses

In an online learning environment, student’s learning motivation and engagement in the learning process are important for their success (Wang et al., 2013). SDL skills are related to the cognitive presence (Garrison, 2003), which, in turn, supports knowledge construction in the online learning process (Hartley & Bendixen, 2001). Considerable research has found that SDL positively influences online learners’ academic achievement (Broadbent & Poon, 2015; Broadbent, 2017; Richardson et al., 2012; Wang et al., 2013). The effects of SDL on online learning have also been confirmed in mobile online learning (Zheng et al., 2018) and collaborative online learning (Kuo et al., 2015). These findings indicate that SDL is pivotal to the success of learning in many different types of online learning (Serdyukov & Hill, 2013).

Given the importance of SDL, online learners should have the skills to plan, monitor, and manage their learning (Ally, 2004). Lin and Hsieh (2001) and Owston (1997) asserted that to be successful in an online learning environment, learners should have the ability to set their learning goals and pace. Therefore, it is critical for online learners to be responsible for controlling their learning (Hartley & Bendixen, 2001; Hsu & Shiue, 2005), follow course schedules (Discenza et al., 2003), be good at self-management (Hill, 2002; Roper, 2007), and actively participate in class activities (Garrison et al., 2004).

### Self-directed learning in MOOCs

MOOCs’ open access offers new learning opportunities for learners around the world for free or at a low cost from universities (Veletsianos et al., 2015), non-profit organizations (Jagannathan, 2015; Zhang et al., 2020), and corporate entities (Bersin, 2013). More than 11,000 MOOCs have been offered by more than 900 universities around the world (Shah, 2019). MOOCs provide an opportunity for learners to gain knowledge and skills (e.g., Barak et al., 2016; Barak & Watted, 2017; Evans et al., 2016; Hew & Cheung, 2014) using diverse delivery modes (e.g., instructor-led and self-paced MOOCs) (Zhu & Bonk, 2019).

MOOCs are different from traditional online courses in terms of the purpose of enrollment, the number of enrolled learners, open access to content, and how students and instructors interact. The average number of learners enrolled in a MOOC is 8000 (Chuang & Ho, 2016), which is much larger than traditional online courses. Given this low instructor-learner ratio, the interaction between instructors and students is very limited. MOOCs also have low completion rates (Jordan, 2013; Lewin, 2012; Reich, 2014), ranging from 7 to 10% (Daniel, 2012; Jordan, 2014).

The critical factors impacting learners’ success in online courses include self-direction, responsibility, and motivation (Grow, 1991; Schrum & Hong, 2002) as well as learners’ cognitive and metacognitive performance (Barnard et al., 2008; Kitsantas et al., 2008; Zimmerman, 1989, 2008). Some researchers have recently examined how to improve these SDL skills in MOOC learners. For example, the Zhu (2021) explored how to enhance MOOC learners’ self-management skills, such as how to promote learning goals, time management, resources and support management, and navigating in MOOCs. Similarly, Onah et al. (2021) explored learners’ self-directed learning abilities in MOOCs and found that goal setting and time management were highly related to their self-regulation skills. However, more work is needed to investigate the impact of these SDL factors on MOOC learners’ successful learning (El-Gilany & Abusaad, 2013; Kop & Fournier, 2010; Terras & Ramsay, 2015; Zhu et al., 2020).

### Learning strategies in MOOCs

Much research has examined the effects of learning strategies on learning outcomes (Alario-Hoyos et al., 2017; Halawa et al., 2014; Littlejohn & Milligan, 2015; Schunk, 2005). A learning strategy refers to “any thoughts, behaviors, beliefs or emotions that facilitate the acquisition, understanding or later transfer of new knowledge and skills” (Weinstein et al., 2000, p. 227). Learning strategies include self-regulated learning (SRL) (Schunk, 2005; Zimmerman, 2002), which is the process that students initiate and maintain cognitive activities towards achieving their learning goals (Zimmerman, 1989). From an SDL perspective, students are expected to control and regulate their own learning using various strategies, such as cognitive, meta-cognitive, and learning resource usage strategies, leading to successful learning outcomes (Pintrich et al., 1993; Zimmerman, 2002). SRL is a sub-concept of SDL (Loyens et al., 2008; Saks & Leijen, 2014). Lin et al. (1999) explained the importance of SDL, especially in a technology-enhanced learning environment. Several studies have also shown that SRL is a reliable predictor of students’ learning outcomes in online learning environments (Halawa et al., 2014; Kizilcec et al., 2017; Lin et al., 2017; Littlejohn & Milligan, 2015).

MOOC learners face unique challenges when learning on their own in an online MOOC environment. Many MOOC learners seem to use inappropriate or insufficient learning strategies (Winne & Jamieson-Noel, 2003) or they do not leverage learning resources to support their learning (Ellis et al., 2005; Lust et al., 2013). Lust et al. (2013) investigated learners’ capacity to use learning resources to support their learning and found that only 3% effectively leveraged the learning resources. They also struggle to finish the courses. The substantially low completion rate of MOOCs, ranging from 7 to 10% (Daniel, 2012; Jordan, 2014), demonstrates the significance of self-regulation for MOOC learners. Littlejohn et al. (2016) explained that this lack of self-regulation is partially due to the restricted interaction with instructors and peers in the MOOC learning environment. Pintrich and de Groot (1990), Zimmerman (1990), and Kim et al. (2019) have also emphasized the importance of self-directed learning strategies in open resource education because successful learners are motivated to use self-regulatory strategies, including planning, monitoring, and adaptation.

Alario-Hoyos et al. (2017) also found that MOOC learners need time management skills to improve self-regulation based on the results of their study with over 6000 MOOC learners. To address time management skills, Yen et al. (2018) developed a self-regulated digital learning framework to facilitate self-regulated learning in online learning environments. The framework has eight features: (1) learning plans (e.g., goal settings or time management); (2) recording and sharing about learning progress; (3) assessment (e.g., reflection, learning outcomes); (4) human feedback; (5) machine feedback; (6) visualization (e.g., a concept map); (7) scaffolding/prompts; and (8) agents. These learning strategies related to self-regulated learning are expected to enhance learning achievement in an online learning environment. Based on the significance of SDL for success in MOOC learning, this study investigates the relationship among motivation, self-monitoring, self-management, and the use of learning strategies.

## Methods

**Fig. 2:**
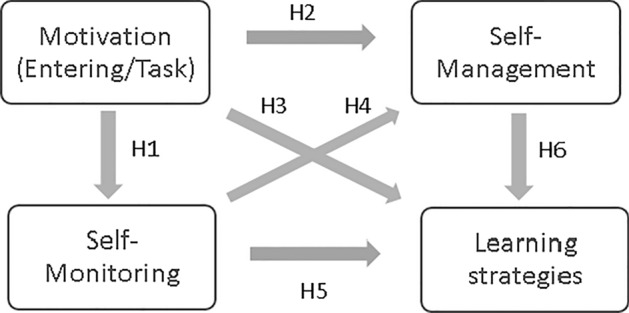
The research model of this study

The theoretical framework of this study is Garrison’s (1997) SDL model, which explains that motivation affects self-monitoring and self-management. Self-monitoring and self-management influence each other. SDL is expected to affect the use of learning strategies of MOOC learners. Thus, this study examines the relationship among motivation, self-monitoring, and self-management and their effects on learning strategies in MOOC learning environments (see Fig. 2). The research question guiding this study is, “To what extent do MOOC learners’ motivation, self-monitoring, and self-management skills predict their use of learning strategies?” Six hypotheses were tested for this study:

*H1*: Motivation positively affects self-monitoring.

*H2*: Motivation positively affects self-management.

*H3*: Motivation positively affects learning strategies.

*H4*: Self-monitoring positively affects self-management.

*H5*: Self-monitoring positively affects learning strategies.

*H6*: Self-management positively affects learning strategies.

### Participants

The participants of this study were MOOC learners who were enrolled in three MOOCs. The first course was a Duke University physiology course with 265,107 students enrolled in Coursera. It took students approximately 31 h to complete the 10-week course. The course videos were in English with Simplified Chinese subtitles. It was rated 4.7/5 by 2224 participants from the beginning of the course offering until February 2020, when this study’s data collection was completed. The second course was an Arizona State University English course with 36,746 enrolled students in Coursera. It took students approximately five hours per week for six months to complete the course. Students watched course videos recorded in English, and students selected subtitles in Arabic, Ukrainian, Simplified Chinese subtitles, Portuguese, Russian, Spanish, Persian, or Tamil. It was rated 4.9/5 by 14,135 participants from the beginning of the course offering until February 2020. The third course was a math course in FutureLearn offered by the Davidson Institute of Science Education in Israel. It took students approximately four hours per week for three weeks to complete the course.

An optional link to an online survey (see Appendix) was inserted into the pages of three MOOCs in the physiology, English, and math courses from November 2018 to February 2020. A total of 470 survey responses were received from students in the three MOOCs. Although the response rate is low, it provides a representative cross-section of enrolled students. The demographic information is presented in Table 1.

**Table 1:** Demographic information

Category	Sub-categories	Numbers (percentage)
Gender	Male	40.2% (N = 189)
	Female	59.1% (N = 278)
Education	High schools	24.7% (N = 116)
	Bachelor’s degree	36.6% (N = 172)
	Master’s degree	22.33% (N = 105)
	Doctoral degrees	8.7% (N = 41)
	Others	7.7% (N = 36)
Employment	Full-time employees	36.4% (N = 171)
	Currently unemployed	25.5% (N = 120)
	Part-time employees	14.3% (N = 67)
	Others (e.g., retired, between jobs, and others)	23.9% (N = 112)
Students (42.7%)	Full-time students	30.6% (N = 144)
	Part-time students	12.1% (N = 57)

The previous MOOC experience of the survey participants ranged from none to more than five courses: none (N = 136, 28.9%), one (N = 96, 20.4%), two (N = 54, 11.5%), three (N = 38, 8.1%), four (N = 19, 4.0%), and five or more (N = 127, 27.0%).

### Instruments

The online survey had four demographic questions and 33 5-point Likert scale questions. The demographic information asked about (1) gender, (2) educational level, (3) current employment status, and (4) MOOC learning experience. The primary variables of this study are self-management (9 items), motivation (i.e., desire for learning) (8 items), and self-monitoring (i.e., self-control) (9 items). These variables, as self-directed learning scales, were developed by adopting instruments from Fisher and King (2010) and Williamson (2007) to MOOC learning environments. Although there are diverse instruments to measure individual variables such as motivation, Fisher and King’s (2010) instrument has been verified to specifically measure SDL as a whole. Learning strategies items measured the learners’ perceptions of discussion, peer-assessment, simulations, interactive technologies, and interaction with instructors. Williamson’s scale originally included 12 items; however, some of the items were excluded because they were not applicable to a MOOC learning environment (e.g., “1 find 'role play' is a useful method for complex learning” or “1 find learning from case studies useful”).

Example statements of self-management are “I set strict time frames for learning in this MOOC” and “I am disorganized while learning in this MOOC” (reversed coded). Sample items of motivation include “I enjoy learning new information through this MOOC” and “I have a need to learn from this MOOC.” Example questions of self-monitoring include “I am responsible for my own learning in this MOOC” and “I am aware of my own limitations when I take this MOOC.” The items for learning strategies (7 items) were developed based on Williamson’s (2007) scale of learning strategies in the self-directed learning process. Learning strategies questions included the ability to choose appropriate learning strategies and transfer of knowledge, such as “I participated in course discussions in this MOOC” and “I am able to relate the knowledge I learned in MOOCs with my work or life.”

The reliability of the variables captured on the questionnaire is in Table 2 with Cronbach’s alphas and related information for the four latent variables. Cronbach’s alphas for the latent variables were higher than 0.70, except for learning strategies. While Cronbach’s alphas for learning strategies were 0.602 in this study, Williamson’s (2007) Cronbach’s alphas were 0.73 for the original scale. Nunnally and Bernstein (1994) suggest that Cronbach's alpha coefficients should be higher than.70. However, Nunnally’s (1967) original work and other researchers (i.e., (Sirakaya-Turk et al., 2017; Ho, 2006) suggested that Cronbach’s alpha coefficient above 0.6 is also acceptable for explanatory studies.

**Table 2:** Research instruments

Variables	Number of items	Cronbach’s alpha	Reference
Self-management	9	.76	Fisher and King (2010) and Williamson (2007)
Self-monitoring	9	.80	Fisher and King (2010) and Williamson (2007)
Motivation	8	.71	Fisher and King (2010) and Williamson (2007)
Learning strategies	7	.60	Williamson (2007)

### Data analysis

To examine the hypotheses in this study, we applied structural equation modeling (SEM), a multivariate analysis method consisting of confirmatory factor analysis and path analysis. SEM is used to examine the relationship between latent constructs and measurement variables (Kline, 2010). Considering the number of measurement variables (33 items) and sample size (n = 470), items were parceled into two bins to achieve better modeling results based on the results of exploratory factor analysis (Little et al., 2002; Matsunaga, 2008). Harman's single-factor test was conducted to detect common method bias. The total variance for a single factor was 21.90%, indicating no common method variance in the data (Podsakoff et al., 2003).

The analysis was conducted with SPSS (Version 27.0) and Amos (Version 26.0). To assess discrepancies between the proposed model and the data, we used several fit indices for analyses: comparative fit index (CFI), Tucker-Lewis index (TLI), root mean square error of approximation (RMSEA), standardized root mean square residual (SRMR), and a chi-square test. CFI and TLI values greater than 0.90 are considered a good fit between a proposed model and the data. In terms of RMSEA, a value of 0.05 indicates a close fit, 0.08 is a fair fit, and 0.10 is a marginal fit (Browne & Cudeck, 1993; MacCallum et al., 1996). For SRMR, Hu and Bentler’s (1999) cutoff value is 0.08 for SRMR.

Confirmatory factor analysis (CFA) was performed using maximum likelihood prior to testing the hypotheses (see Table 3). Based on the convergent validity guidelines by Fornell and Larcker (1981) and Hair et al. (2006), factor loading values for individual items should be higher than 0.5. The CFA results for all factor loadings were over 0.6, and the measurement model indicated a good fit for the data. Convergent validity was examined using average variance extracted (AVE) and composite reliability (CR). The AVE values were over 0.5, and all CR values of the constructs were over 0.7. The results confirmed that the overall CFA, including factor loadings, AVE, and CR values of the data, were all satisfactory.

**Table 3:** Results of confirmatory factor analysis

Latent variable	Measurement variable	Factor loading (> .5)	AVE (> .5)	CR (> .7)
Motivation	Motivation 1	.65	.73	.85
	Motivation 2	.72		
Self-monitoring	Self-monitoring 1	.97	.85	.92
	Self-monitoring 2	.55		
Self-management	Self-management 1	.65	.67	.80
	Self-management 2	.75		
Learning strategies	Learning strategies 1	.54	.74	.85
	Learning strategies 2	.85		

To assess the discriminant validity, the square root of the correlations for each latent variable and AVE value were compared. The AVE values for the latent variables, as shown in Table 4, were greater than the square root of the correlation. The results indicated that the discriminant validity was acceptable.

**Table 4:** Discriminant validity assessment

Measures	Motivation	Self-monitoring	Self-management	Learning strategies	AVE	CR
Motivation (ρ2)	–	.66 (.43)	.60 (.36)	.63 (.40)	.73	.85
Self-monitoring (ρ2)		–	.66 (.43)	.50 (.25)	.85	.92
Self-management (ρ2)			–	.48 (.23)	.67	.80
Learning strategies (ρ2)				–	.74	.85

The statistical significance of the path coefficient among the latent variables in the research model was examined using the fitness index.

As indicated in Table 5, the research model indicated a fair fit to the data (χ^2^ = 46.80; df = 14; CMIN/df = 3.34; TLI = 0.93; CFI = 0.97; SRMR: 0.04; RMSEA = 0.07) (Browne & Cudeck, 1993; MacCallum et al., 1996). The results imply that the hypothesized model is fair to explain the relationship among the variables in data.

**Table 5:** Results of the fitness of the research model (n = 470)

	*χ*^*2*^	*p*	*df*	*TLI*	*CFI*	*SRMR*	*RMSEA* (90% confidence interval)
Structural model	46.80	.001	14	.93	.97	.04	.07 (.05 ~ .08)
Fit criteria				> .90	> .90	< .08	< .08

## Results

### Descriptive analysis

Descriptive data for the four latent variables (i.e., motivation, self-monitoring, self-management, and learning strategies) are summarized in Table 6. The correlations between the four latent variables are statically significant at p < . 001.

**Table 6:** Descriptive data

Latent variables	Mean	SD	Correlation			
			1	2	3	4
Motivation	3.99	.48	1			
Self-monitoring	4.14	.45	.51**	1		
Self-management	3.79	.54	.40**	.53**	1	
Learning strategies	3.58	4.79	.50**	.36**	.33**	1

**p < .001; *p < .05

Table 7 summarizes the descriptive statistics, including the means, standard deviations, and correlations among the measurement variables. The skewness and kurtosis of each measurement were computed as an indicator of normal distribution. The minimum/maximum values were from − 2 to 2, so the normal distribution assumption was considered tenable (George & Mallery, 2010).

**Table 7:** Correlation between measurement variables

Variables	1	2	3	4	5	6	7	8
Motivation 1	1	.47**	.44**	.31**	.26**	.28**	.18**	.32**
Motivation 2		1	.43**	.21**	.23**	.37**	.30**	.40**
Self-monitoring 1			1	.53**	.46**	.45**	.22**	.42**
Self-monitoring 2				1	.29**	.27**	.09**	.18**
Self-management 1					1	.51**	.18**	.21**
Self-management 2						1	.28**	.36**
Learning strategies 1							1	.46**
Learning strategies 2								1
Mean	4.49	3.83	4.04	4.46	4.08	3.59	3.50	3.65
SD	.53	.60	.52	.52	.74	.65	.63	.51
Skewness	− .99	− .02	.05	− .53	− .92	.03	.19	.00
Kurtosis	1.07	− .17	− .45	− .46	1.26	.18	− .32	.71

**p < .001; *p < .05

**Fig. 3:**
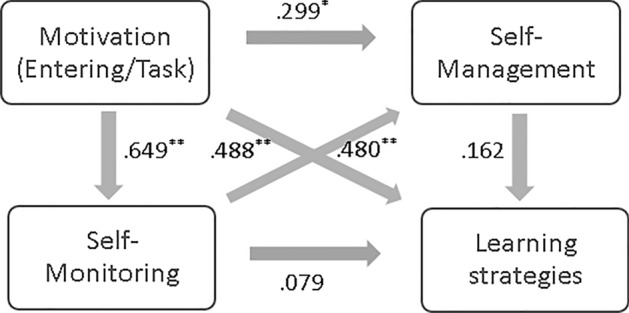
The results of hypothesis testing

To test the six hypotheses, we examined the statistical significance of the path coefficient among the variables. The results indicated that H1, H2, H3, and H4 were accepted (*t* > 1.96, *p* < 0.05) as displayed in Table 8. Motivation had a significant influence on self-monitoring (*β* = 0.65, *t* = 6.57), self-management (*β* = 0.30, *t* = 2.90), and learning strategies (*β* = 0.49, *t* = 4.01). Thus, H1, H2, and H3 were supported. Self-monitoring positively influenced self-management (*β* = 0.46, *t* = 4.94) but not learning strategies (*β* = 0.08, *t* = 0.37), which indicated that H4 was supported, and H5 was rejected. Self-management did not influence the use of learning strategies (*β* = 0.16, *t* = 1.67), indicating that H6 was not supported. The results of testing the research model are shown in Fig. 3.

**Table 8:** Path coefficient estimates

Hypothesis	B	β	SE	t-value
H1: Motivation → Self-monitoring	.43	.65**	.07	6.57
H2: Motivation → Self-management	.35	.30*	.12	2.90
H3: Motivation → Learning strategies	.39	.49**	.10	4.01
H4: Self-monitoring → Self-management	.82	.46**	.17	4.94
H5: Self-monitoring → Learning strategies	.10	.08	.89	.37
H6: Self-management → Learning strategies	.11	.16	.10	1.67

**p < .001; *p < .05

As shown in Table 9, indirect effects of motivation on self-management through self-monitoring were observed at *p* < 0.05. However, the indirect effects of motivation and self-monitoring on learning strategies were not statistically significant.

**Table 9:** Direct and indirect effects

Hypothesis	Total Effects	Direct Effects	Indirect effects
H1: Motivation → Self-monitoring	.65*	.65*	–
H2: Motivation → Self-management (through self-monitoring)	.60*	.30*	.30*
H3: Motivation → Learning strategies (through self-management)	.64*	.49*	.15
H4: Self-monitoring → Self-management	.48*	.48*	–
H5: Self-monitoring → Learning strategies (through self-management)	.15	.08	.07
H6: Self-management → Learning strategies	.16	.16	–

**p < .001; *p < .05

## Discussion

The importance of MOOCs as a learning paradigm has increased with rising student enrollments. MOOC learners are expected to be self-directed learners and to adopt appropriate learning strategies for their learning outcomes. Considering the open-access characteristics of MOOCs, the intention and motivation of MOOC learners are diverse (e.g., auditing students, sporadic students, serious learners who complete MOOCs). Kovanović et al. (2019) classified MOOC learners into three categories based on their study strategies: limited users, selective users, and broad users. This study aimed to understand the relationship among motivation, self-monitoring, and self-management and their effects on the use of MOOC learners' learning strategies.

This study applied Garrison’s SDL model to a MOOC learning environment to test the six hypotheses. The results indicated that motivation positively influenced self-monitoring (H1) and self-management (H2), and self-monitoring positively affected self-management (H4). The findings also indicated that motivation facilitated self-monitoring, self-management, and the adoption of learning strategies. Among the three components of Garrison’s SDL model, only motivation had positive effects on learning strategies in SDL. The results of this study confirmed the research findings by Kovanović et al. (2019) and Alario-Hoyos et al. (2017). In particular, the current study confirmed Kovanović et al.’s (2019) findings that MOOC learners’ intention and motivation to take a MOOC course determine their use of learning strategies. Many researchers have examined the importance of learners’ motivation and learning strategies in MOOCs. Alario-Hoyos et al. (2017) found that MOOC learners were generally motivated, and learners’ learning strategies could be enhanced. Building on these previous findings, this study revealed that motivation could predict students’ learning strategies such as online interactions with instructors and teaching assistants, participation in online discussions, assessment, and transferring knowledge to their work and life (H3).

Learning strategy refers to factors that support the acquisition, understanding, and transferring of knowledge and skills. Learning strategies can predicate online students’ learning outcomes (Halawa et al., 2014; Kizilcec et al., 2017; Littlejohn & Milligan, 2015) and can be an effective approach for self-regulated learning (SRL) strategies (Schunk, 2005; Zimmerman, 2002). In SDL, students should use diverse learning strategies to direct, control, and regulate their own learning (Pintrich et al., 1993; Zimmerman, 2002). Both SRL and learning strategies are required in education, especially in a technology-enriched learning environment (Lin et al., 1999). Researchers have reported that learning strategies are reliable predictors of students’ learning outcomes in online learning environments (Halawa et al., 2014; Kizilcec et al., 2017; Littlejohn & Milligan, 2015). Thus, research is needed to examine learners’ diverse motivations and strategies to improve learners’ motivation and enhance their learning strategies. MOOC instructors should examine students’ initial motivation as the learning initiative and regularly monitor their task motivation during the program to check their learning persistence. This study also demonstrated that motivation is critical to learning in MOOCs in terms of encouraging learners to adopt learning strategies for successful learning. In addition, the results showed that motivation is a prerequisite element in SDL (Fournier et al., 2014). In particular, this study found that motivation had indirect effects on self-management through self-monitoring as well as direct influence on self-management.

In the current study, self-monitoring and self-management did not influence the adoption of learning strategies. Therefore, we do not expect that those who have self-monitoring or self-management skills will adopt effective learning strategies for their learning (H5, H6). More research should be conducted to investigate the relationship between self-monitoring, self-management, and learning strategies. One plausible reason for this research finding could be explained by the items measuring learning strategies. Considering the importance of instructional methods and media in MOOCs, we included questions in the measurement scale of learning strategies asking about students’ perceptions of the instructional methods and media as well as their cognitive or meta-cognitive skills, (i.e., abilities to choose learning strategies and the capabilities to transfer knowledge). The findings may have been influenced by combining these two different levels of learning strategies, the perceptions of instructional methods and media, and cognitive or meta-cognitive skills for learning.

## Limitations

The data were collected from three MOOCs which were taught in English (with selected subtitles), thus limiting the generalizability of our research findings to students who enrolled in MOOCs in local languages (i.e., other than English) in other countries. More participants from diverse MOOCs may increase the generalizability of this study. Second, we did not distinguish between students from different subject areas or the various lengths of the MOOCs. It would be interesting for future studies to explore whether the subject area or the length of a MOOC influences students’ self-directed learning skills. Third, the survey participants were self-selected volunteers, which could introduce bias, given that volunteers may have more SDL skills. Fourth, the data of this study were collected from a self-reported questionnaire, and Harman's single-factor test confirmed that there was no common method variance in our data (i.e., total variance for a single factor was 21.90). Therefore, to strengthen and verify the research findings, various data collection methods such as interviews and log data are strongly recommended in the future.

## Conclusion

Coronavirus (COVID-19) has given students who were used to learning in traditional classrooms opportunities to experience online learning as an alternative learning method. Online learning, including MOOCs, is expected to become more widespread across the globe. MOOCs allow students more autonomy, flexibility, and independence in learning. However, it requires that students are self-directed learners and can choose and adopt appropriate learning strategies for successful learning outcomes. The motivation of MOOC learners varies and influences their learning outcomes through self-directed learning skills and learning strategies. Kovanović et al. (2019) noted that providing learning tools and activities is not sufficient to enhance learning for MOOC students. Researchers and instructors need to pay more attention to MOOC learners’ motivation and encourage them to adopt appropriate learning strategies for successful learning.

## Appendix: Questionnaires used in the study

**Table:**

Variables	Items
Self-management	1. I prefer to plan my own learning in this MOOC
	2. I am self-disciplined while learning in this MOOC
	3. I have good management skills (e.g., time, learning resources, etc.) in this MOOC
	4. I set specific times to study in this MOOC
	5. I set strict time frames for learning in this MOOC
	6. I am able to keep my learning routine in this MOOC separate from my other commitments
	7. I can apply a variety of learning strategies in this MOOC
	8. I am disorganized while learning in this MOOC
	9. I am confident in my ability to search for information related to learning content in this MOOC
Motivation	1. I have a need to learn from this MOOC
	2. I want to learn new information through this MOOC
	3. I enjoy learning new information through this MOOC
	4. I enjoy the various challenges of this MOOC
	5. I critically evaluate new ideas in this MOOC
	6. I need to know the deeper reasons of the facts in this MOOC
	7. I learn from my mistakes in this MOOC
	8. When presented with a problem I cannot resolve, I will ask for assistance through different means provided by this MOOC
Self-monitoring	1. I am responsible for my own learning in this MOOC
	2. I am in control of my learning in this MOOC
	3. I have high learning standards when I take this MOOC
	4. I prefer to set my own learning goals in this MOOC
	5. I evaluate my own performance in this MOOC
	6. I have high beliefs in my learning abilities in this MOOC
	7. I can find information related to learning content for myself when I take this MOOC
	8. I am able to focus on answering or solving a problem in this MOOC
	9. I am aware of my own limitations when I take this MOOC
Learning strategies	1. I participated in course discussions in this MOOC
	2. Peer-assessment is effective in this MOOC
	3. Interacting with the instructor or teaching assistant is more helpful than just learning alone in this MOOC
	4. Learning through simulations is helpful in MOOCs
	5. Interactive educational technology (e.g., automatic feedback, online games, learner polling, discussion forums, etc.) enhances my learning in this MOOC
	6. I am able to choose my own learning strategies in this MOOC
	7. I am able to relate the knowledge I learned in MOOCs with my work or life
